# Extended-Spectrum Cephalosporin-Resistant *Salmonella enterica* serovar Heidelberg Strains, the Netherlands^1^

**DOI:** 10.3201/eid2207.151377

**Published:** 2016-07

**Authors:** Apostolos Liakopoulos, Yvon Geurts, Cindy M. Dierikx, Michael S.M. Brouwer, Arie Kant, Ben Wit, Raymond Heymans, Wilfrid van Pelt, Dik J. Mevius

**Affiliations:** Wageningen University, Lelystad, the Netherlands (A. Liakopoulos, Y. Geurts, C.M. Dierikx, M.S.M. Brouwer, A. Kant, D.J. Mevius);; Netherlands Food and Consumer Product Safety Authority, Utrecht, the Netherlands (B. Wit, R. Heymans);; National Institute for Public Health and the Environment, Bilthoven, the Netherlands (W. van Pelt);; Utrecht University, Utrecht (D.J. Mevius)

**Keywords:** *Salmonella enterica* serovar Heidelberg, extended-spectrum cephalosporins, AmpC, IncI1 plasmids, the Netherlands, bacteria, antibacterial agents, Salmonella infections, antimicrobial resistance

## Abstract

Extended-spectrum cephalosporin-resistant *Salmonella enterica* serovar Heidelberg strains (JF6X01.0022/XbaI.0251, JF6X01.0326/XbaI.1966, JF6X01.0258/XbaI.1968, and JF6X01.0045/XbaI.1970) have been identified in the United States with pulsed-field gel electrophoresis. Our examination of isolates showed introduction of these strains in the Netherlands and highlight the need for active surveillance and intervention strategies by public health organizations.

*Salmonella enterica* serovar Heidelberg is among the most prevalent causes of human salmonellosis in the United States and Canada but has been reported infrequently in Europe (*1**–**3*). Although most nontyphoidal *Salmonella* infections are self-limiting and resolve within a few days, *Salmonella* ser. Heidelberg tends to provoke invasive infections (e.g., myocarditis and bacteremia) that require antimicrobial drug therapy (*4*). To treat systemic nontyphoidal *Salmonella* infections, third-generation cephalosporins are preferred drugs for children or for adults with fluoroquinolone contraindications (*5*). Resistance to third-generation cephalosporins is increasing in *S. enterica* infections, mainly because of production of plasmid-mediated extended-spectrum or AmpC β-lactamases (*6*).

Resistance to extended-spectrum cephalosporins (ESCs) among *Salmonella* Heidelberg strains found in human infections, food-producing animals, and poultry meat indicates zoonotic and foodborne transmission of these strains and potential effects on public health (*7*,*8*). Unlike in Canada and the United States, few ESC-resistant *Salmonella* Heidelberg strains have been documented in Europe (*9**–**13*). However, increased occurrence of ESC resistance in *S. enterica* infections and decreased susceptibility to fluoroquinolones compromise the use of these drugs and constitute a serious public health threat (*6*,*14*).

Few data are available regarding prevalence of ESC-resistant *Salmonella* Heidelberg isolates in Europe, their underlying antimicrobial drug resistance gene content, and genetic platforms (i.e., plasmids and insertion sequence [IS] elements) associated with resistance genes. We attempted to determine the occurrence and molecular characteristics of *Salmonella* Heidelberg isolates recovered from human patients, food-producing animals, and poultry meat in the Netherlands during 1999–2013.

## The Study

During 1999–2013, the Netherlands National Institute of Public Health and the Environment collected 437 *Salmonella* Heidelberg isolates from human infections (n = 77 [17.6%]), food-producing animals (n = 138 [31.6%]), poultry meat (n = 170 [38.9%]), and other sources (n = 52 [11.9%]). From this collection, we selected 200 epidemiologically unrelated isolates for further analysis (Table; Technical Appendix).

**Table:** Characteristics of *Salmonella enterica* serovar Heidelberg isolates recovered from human infections, food-producing animals, poultry meat, and other sources, the Netherlands, 1999–2013*

Source	1999–2001	2002–2004	2005–2007	2008–2010	2011–2013
Human infections					
No. isolates studied	13	10	22	23	15
Resistance phenotypes (no.)	Amp (1), AmpCol (1), AmpSmxTmpStr (1), AmpTetSmxTmpStr (1), SmxStr (1), Str (5), TetSmxTmpStr (1), WT (2)	AmpSmxStr (1), AmpTetSmx (1), SmxStr (3), Str (1), TetSmxStr (1), WT (3)	AmpFotTazStr (1), AmpSmxTmpNalCip (1), AmpTet (1), NalCip (2), SmxStr (1), Tet (1), TetSmxNalCip (1), WT (14)	ChlCol (1), Col (10), Str (1), StrCol (5), TetCol (1), TetNalCip (1), TetSmxTmp StrCol (1), TetStr KanCol (1), TetStr SmxCol (1), WT (1)	Col (1), Str (3), TetSmxStr (2), TetSmxTmp (1), WT (8)
No. ESCR isolates	0	0	1	0	0
Food-producing animals					
No. isolates studied	5	16	5	7	13
Resistance phenotypes (no.)	NalCip (1), WT (4)	Amp (3), AmpSmxTmpNalCipStr (2), AmpStr (2), NalCip (5), SmxStrTmp (1), WT (3)	AmpTetSmxTmpNalCip (1), WT (4)	AmpCol (1), AmpFotTazNalCip (1), AmpFotTazTetSmx GenStrKanCol (1), Col (4)	AmpCol (1), AmpFotTazTetSmx (1), AmpFotTazTet SmxNalCip (4), Col (2), TetSmxNalCip (2), TetSmxNal CipGenStrKan (1), WT (2)
No. ESCR isolates	0	0	0	2	5
Poultry meat					
No. isolates studied	3	3	15	6	40
Resistance phenotypes (no.)	AmpTetSmxTmpNalCipStr (1), SmxTmpStr (1), WT (1)	AmpSmxStr (1), WT (2)	NalCip (3), SmxCipGen (1), SmxGen (1), SmxTmpNalCip (1), TetSmxTmp (1), WT (8)	AmpFotTaz (1), AmpFotTazSmxTmp ChlStrCol (1), AmpFotTazStrCol (1), Col (2), NalCipCol (1)	AmpFotTazTetSmx NalCip (26), AmpFotTazTetSmx NalCipCol (1), AmpFotTazTetSmx NalCipGenStrKan (1), AmpFotTaz TetSmxNalCipStr (6), AmpFotTazTetSmx TmpNalCipChl (1), Col (2), TetSmx NalCip (1), TetSmx NalCipGenStr (1), TetSmxNalCipStr (1)
No. ESCR isolates	0	0	0	3	35
Other					
No. isolates studied	0	1	0	6	4
Resistance phenotypes (no.)		WT (1)		Col (2), NalCipCol (1), Str (1), StrCol (2)	AmpFotTazTetSmx NalCip (1), NalCipCol (1), Str (1), TetSmxNal CipGenStr (1)
No. ESCR isolates	0	0	0	0	1

*Amp, ampicillin; Cip, ciprofloxacin; Chl, chloramphenicol; Col, colistin; ESCR, extended-spectrum cephalosporin-resistant; Fot, cefotaxime; Gen, gentamicin; Kan, kanamycin; Nal, nalidixic acid; Smx, sulfamethoxazole; Str, streptomycin; Taz, ceftazidime; Tet, tetracycline; Tmp, trimethoprim; WT, wild type.

MICs for antimicrobial agents were determined with the broth microdilution method (online Technical Appendix) and showed a higher frequency of multidrug non–wild-type susceptibility phenotype in isolates from poultry meat (n = 44 [68.8%]) than in isolates from food-producing animals (n = 14 [31.8%]) and human infections (n = 16 [19.5%]). Most human infections exhibited wild-type MICs to most antimicrobial agents tested (Table).

**Figure 1:**
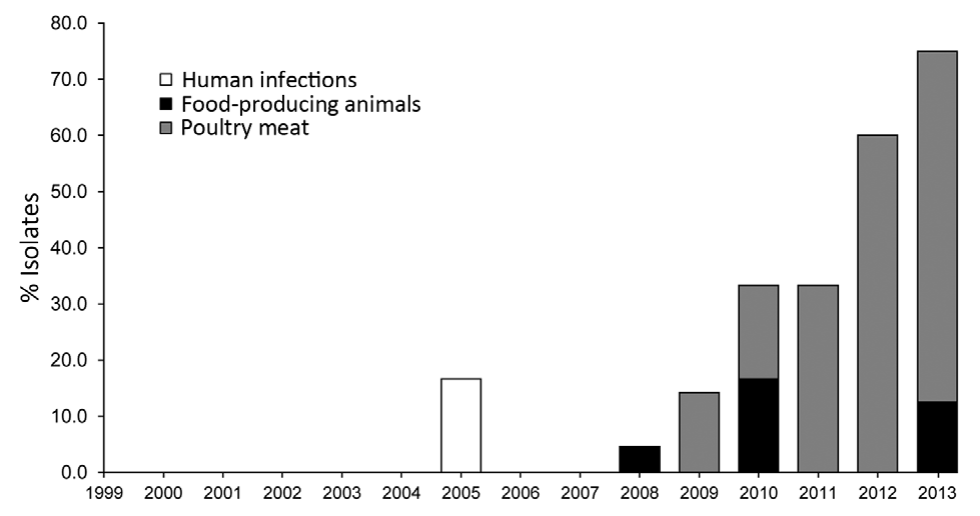
Occurrence of extended-spectrum cephalosporin-resistant *Salmonella* *enterica* serovar Heidelberg isolates, the Netherlands, 1999–2013.

Of the 200 *Salmonella* Heidelberg isolates in the study, 47 (23.5%) were ESC resistant. ESC resistance in *Salmonella* Heidelberg isolates increased from 33.3% in 2011 to 60.0% in 2012 to 75.0% in 2013, after which *Salmonella* Heidelberg was the predominant serotype in ESC-resistant *Salmonella* isolates in the Netherlands (Figure 1).

These isolates showed MICs for cefotaxime and ceftazidime of 2 to >4 mg/L and 4 to >16 mg/L, respectively; non–wild-type susceptibility to fluoroquinolones was 87.2%. The emergence of isolates with decreased susceptibility to these first-line antimicrobial drugs limits effective treatment options for potential human infections.

**Figure 2:**
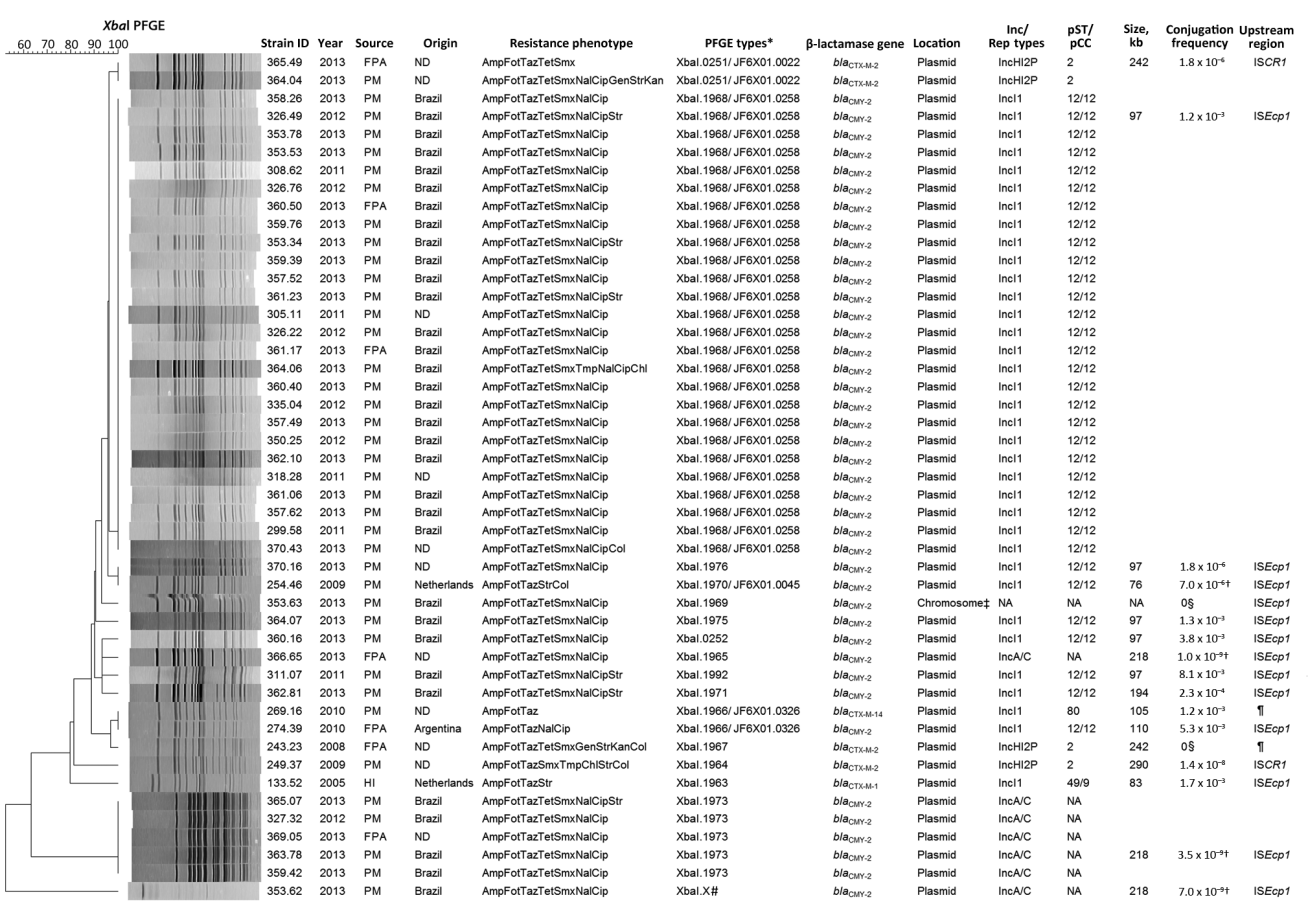
Characteristics of extended-spectrum cephalosporin-resistant *Salmonella* *enterica* serovar Heidelberg isolates, the Netherlands, 1999–2013. The dendrogram was generated by using BioNumerics version 6.6 (Applied Maths, Sint-Martens-Latem, Belgium) and indicates results of a cluster analysis on the basis of *Xba*I–pulsed-field gel electrophoresis (PFGE) fingerprinting. Similarity between the profiles was calculated with the Dice similarity coefficient and used 1% optimization and 1% band tolerance as position tolerance settings. The dendrogram was constructed with the UPGMA method based on the resulting similarity matrix. Amp, ampicillin; Cip, ciprofloxacin; Chl, chloramphenicol; Col, colistin; Fot, cefotaxime; FPA, food-producing animals; Gen, gentamicin; HI, human infection; Kan, kanamycin; Nal, nalidixic acid; ND, not determined (i.e., refers to isolates recovered in the Netherlands but with unknown origin of the sample); pCC, plasmid clonal complex; PM, poultry meat; pST, plasmid sequence type; Smx, sulfamethoxazole; Str, streptomycin; Taz, ceftazidime; Tet, tetracycline; Tmp, trimethoprim. *Pattern numbers assigned by The European Surveillance System molecular surveillance service of the European Centre for Disease Prevention and Control database and corresponding pattern numbers from the PulseNet database (http://www.cdc.gov/pulsenet/index.html). †Results refer to the conjugation frequencies during filter-mating experiments. ‡Chromosomal location confirmed by I-*Ceu*I PFGE of total bacterial DNA, followed by Southern blot hybridization. §No transconjugants were obtained after liquid and filter-mating experiments, suggesting the presence of nonconjugative plasmids or conjugation frequencies below detection limits. ¶Insertion sequences IS*Ecp1*, IS*CR1*, or IS26 were not found upstream of the extended-spectrum β-lactamase genes for these PFGE types. #This PFGE fingerprint was not submitted to The European Surveillance System molecular surveillance service of the European Centre for Disease Prevention and Control database for name assignment.

ESC typing of the 47 isolates, performed by microarray analysis followed by PCR and sequencing (Technical Appendix), revealed the presence of the *bla*CMY-2 gene in 41 ESC-resistant *Salmonella* Heidelberg isolates that exhibited an AmpC β-lactamase phenotype. The other 6 isolates exhibited an extended-spectrum β-lactamase phenotype and encoded *bla*_CTX-M-2_ (n = 4), *bla*_CTX-M-1_ (n = 1), or *bla*_CTX-M-14_ (n = 1) genes (Figure 2).

We assessed the genetic relatedness of the 47 cephalosporin-resistant *Salmonella* Heidelberg isolates by using the standardized *Xba*I–pulsed-field gel electrophoresis (PFGE) (online Technical Appendix), which identified 2 major PFGE types: *Xba*I.1968 and *Xba*I.1973 (PFGE numbers assigned by the European Centre for Disease Prevention and Control, Solna, Sweden). Of the 47 isolates, 26 (55.3%) belonged to *Xba*I.1968 and 5 (10.6%) belonged to *Xba*I.1973. Forty-one of the isolates were *bla*_CMY-2_ carriers, 31 (75.6%) of which belonged to these 2 PFGE types; 10 (24.4%) were distributed equally among other PFGE types. Six of the 47 isolates were *bla*_CTX-M_ carriers associated with 5 PFGE types (Figure 2). Comparing these isolates with those in the PulseNet database (http://www.cdc.gov/pulsenet/index.html) revealed the introduction of 4 epidemic clones of ESC-resistant *Salmonella* Heidelberg strains in the Netherlands (JF6X01.0022/*Xba*I.0251, JF6X01.0326/*Xba*I.1966, JF6X01.0258/*Xba*I.1968, and JF6X01.0045/*Xba*I.1970). To raise awareness and determine whether related ESC-resistant *Salmonella* Heidelberg isolates had been observed in other European countries, the Epidemic Intelligence Information System (European Centre for Disease Prevention and Control) issued an alert on September 18, 2014.

We successfully transferred plasmids carrying extended-spectrum or AmpC β-lactamases from ESC-resistant *Salmonella* Heidelberg isolates to the recipient *E. coli* DH10B strain (Technical Appendix). PCR-based Inc/Rep typing and multilocus or double-locus sequence typing (ST) of the plasmids revealed that the *bla*_CMY-2_ or *bla*_CTX-M_ genes were located on plasmids for 46 (97.8%) of the 47 isolates. ESC-resistant *Salmonella* Heidelberg isolates encoding *bla*_CMY-2_ on IncI1/ST12 plasmids were associated predominantly with the *Xba*I.1968 (n = 26 [78.8%]) PFGE type; those encoding *bla*_CMY-2_ on IncA/C plasmids were associated with *Xba*I.1973 (n = 5 [71.4%]). Isolates encoding *bla*_CTX-M-2_ on IncHI2P/ST2, *bla*_CTX-M-1_ on IncI1/ST49, and *bla*_CTX-M-14_ on IncI1/ST80 plasmids were associated with *Xba*I.1964, *Xba*I.1963, and *Xba*I.1966, respectively (Figure 2).

The *bla*_CMY-2_ gene was present in 12 different PFGE types and was carried on plasmids of 2 different incompatibility groups (IncI1/ST12 and IncA/C) or on the chromosome. This gene’s diverse genetic background suggests that emergence of the *bla*_CMY-2_–producing *Salmonella* Heidelberg strain in the Netherlands results not only from expansion of a single clone but from multiclonal dissemination of the strain and horizontal transfer of plasmids encoding the *bla*CMY-2 gene. IncI1/ST12 and IncA/C plasmids have been associated with the *bla*CMY-2 gene in *Salmonella* Heidelberg isolates in the United States and Canada (*8*,*15*).

We analyzed a subset of ESC-resistant *Salmonella* Heidelberg isolates to determine the size and conjugation frequency of plasmids carrying extended-spectrum and AmpC β-lactamases. We also assessed a subset of *Salmonella* Heidelberg isolates (n = 17) for each PFGE type, including isolates for each type if they showed variation in extended-spectrum and AmpC β-lactamase genes or in gene location. This assessment sought to detect the upstream presence of resistance genes (*bla*_CTX-M_ and *bla*_CMY_) of frequently encountered insertion sequences (IS*Ecp1*, IS*CR1*, and IS*26*) (Figure 2; Technical Appendix). 

 We attribute the increase of ESC-resistant *Salmonella* Heidelberg isolates in the Netherlands to the frequent occurrence of isolates carrying IncI1/ST12 plasmids encoding *bla*_CMY-2_ in food-producing animals and poultry products imported from Brazil. Isolates from imported poultry products are associated predominantly with PFGE types *Xba*I.1968 and *Xba*I.1973 (Figure 2). A similar introduction of ESC-resistant *Salmonella* Heidelberg strains in Ireland was associated with imported poultry meat from Brazil (R. Slowey, pers. comm.). Although ESC-resistant *Salmonella* Heidelberg strains are rarely reported in Europe, their introduction through imported poultry meat could pose a public health risk; Brazil is among the world’s leading countries for exporting poultry meat.

## Conclusions

Most ESC-resistant *Salmonella* Heidelberg isolates in our study had profiles (*Xba*I.0251, *Xba*I.1966, *Xba*I.1968, and *Xba*I.1970) indistinguishable from those of previous epidemic types (JF6X01.0022, JF6X01.0326, JF6X01.0258, and JF6X01.0045) that caused outbreaks and showed potency for bloodstream infections (*16*). Our identification of clonal clusters shared by ESC-resistant *Salmonella* Heidelberg strains in food-producing animals or poultry meat that can cause human infections underscores the risk for potential zoonotic or foodborne transmission of these strains to humans.

Although we observed a frequent occurrence of ESC-resistant *Salmonella* Heidelberg isolates in poultry products, no human infections linked to these contaminated products have been yet documented in the Netherlands. Nevertheless, the risk of potential zoonotic or foodborne transmission of ESC-resistant *Salmonella* Heidelberg strains highlights the necessity for active surveillance and intervention strategies by public health organizations.

Technical Appendix Detailed methods and materials used in study of extended-spectrum cephalosporin-resistant *Salmonella enterica* serovar Heidelberg strains, the Netherlands.
