# Supplementary material for: Extended-Spectrum Cephalosporin-Resistant Salmonella enterica serovar Heidelberg Strains, the Netherlands
Source: Emerg Infect Dis. 2016 Jul;22(7):1257–61. doi: 10.3201/eid2207.151377 (PMC4918182; doi:10.3201/eid2207.151377)
Supplement: Technical Appendix — Detailed methods and materials used in study of extended-spectrum cephalosporin-resistant Salmonella enterica serovar Heidelberg strains, the Netherlands. [file 15-1377-Techapp-s1.pdf]

Article DOI: http://dx.doi.org/10.3201/eid2207.151377

Extended-Spectrum Cephalosporin-Resistant *Salmonella enterica* serovar Heidelberg Strains, the Netherlands

Technical Appendix

Materials and Methods

Bacterial Strains and Identification

During 1999–2013, the Dutch National Institute of Public Health (RIVM) collected 30,472 *Salmonella* isolates from various surveillance programs on patients with salmonellosis and from farms, slaughterhouses, and retail markets. The isolates originated from human infections (n = 17,363), food-producing animals (n = 6,136), poultry meat (n = 1,260) and other sources (n = 5,713). Using micronitration, RIVM performed serotyping based on somatic (O) and flagellar (H) antigens according to the latest version of the White-Kaufmann-Le Minor scheme (*1*). Recovered *Salmonella* isolates were stored at –80°C in Peptone Broth supplemented with 30% (v/v) glycerol for further analysis.

We selected 200 isolates from the 437 *Salmonella enterica* serovar Heidelberg isolates received at RIVM during 1999–2013. Only the first isolate per patient was included, and to avoid epidemiologically clustered isolates in the selection, only 1 isolate was included per sample type (i.e., human infection, food-producing animal, poultry meat, or others) and origin (i.e., hospital, institute, laboratory, farm, company, or surveillance program) per 14-day period.

Antimicrobial Susceptibility Testing

The susceptibility of the isolates to antimicrobial agents was assessed by broth microdilution, as described by the International Standard Organization (standard 20776–1:2006), by using microtiter trays with a custom-designed, dehydrated panel of antimicrobial drugs (EUMVS, Sensititre, Thermo Fischer, Basingstoke, UK). The antimicrobial agents tested included ampicillin, cefotaxime, ceftazidime, ciprofloxacin, chloramphenicol, colistin, florfenicol, gentamicin, kanamycin, nalidixic acid, streptomycin, sulfamethoxazole, trimethoprim, and tetracycline. *Escherichia coli* strain ATCC 25922 and *Enterococcus*

faecalis strain ATCC 29212 were used as quality controls. For interpretation, we used epidemiologic cutoff values recommended by the European Committee on Antimicrobial Susceptibility Testing (http://mic.eucast.org). Multidrug non–wild-type phenotype was defined as non–wild-type MICs to ≥1 antimicrobial agents from ≥3 antimicrobial classes. Production of extended-spectrum or AmpC β-lactamases was evaluated by a combined disc test that used discs of cefotaxime and ceftazidime with (30/10 μg) and without clavulanic acid (30 μg) and a disc of cefoxitin (30 μg) for all isolates; this process satisfied the phenotypic criteria indicative of extended-spectrum cephalosporinase production, as recommended by the European Centre for Disease Prevention Control (*2*).

Characterization of Resistance Determinants

We assessed the presence of genes conferring the extended-spectrum cephalosporinase-resistant phenotype. DNA was extracted by using the DNeasy Blood and Tissue kit (QIAGEN, Hilden, Germany) according to the manufacturer's recommendations. All isolates putatively producing extended-spectrum or AmpC β-lactamases were screened for a broad spectrum of extended-spectrum and AmpC β-lactamase gene families by using the Check-MDR CT-101 array platform (Check-Points, Wageningen, the Netherlands) according to the manufacturer's recommendations. The presence of extended-spectrum or AmpC β-lactamase genes was confirmed by PCR and subsequent sequencing as described (*3*). The nucleotide and deduced amino acid sequences were compared with sequences in the Lahey clinic database (http://www.lahey.org/Studies).

Clonal Analysis

All isolates carrying extended-spectrum or AmpC β-lactamases (n = 47) and isolates randomly selected on the basis of year and source of isolation (n = 64) were analyzed for genetic relatedness by pulsed-field gel electrophoresis (PFGE) of *Xba*I-digested genomic DNA by using a CHEF DR-III apparatus (Bio-Rad Laboratories, Hercules, CA, USA), according to the standardized protocol of PulseNet (*4*). *Xba*I-digested genomic DNA from *S. enterica* ser. Braenderup strain H9812 was used as a molecular reference marker (*5*). Image normalization and construction of similarity matrices were carried out by using BioNumerics, version 6.6 (Applied Maths, Sint-Martens-Latem, Belgium). Bands were assigned manually, and dendrograms were generated by employing the Unweighted Pair Group Method with Arithmetic mean based on the Dice similarity index, by using 1% optimization and 1% band tolerance as position tolerance settings.

PFGE fingerprints of the isolates were submitted to The European Surveillance System molecular surveillance service of the European Centre for Disease Prevention and Control database, which assigned pattern names. PFGE fingerprints were subsequently compared with those from the PulseNet database. An alert was issued on September 18, 2014, through the European Epidemic Intelligence Information System for the Food and Waterborne Diseases and Zoonoses network to raise awareness and determine whether related extended-spectrum cephalosporinase-resistant *S. enterica* ser. Heidelberg isolates had been observed in other member countries of the European Epidemic Intelligence Information System.

Plasmid Analysis

The replicon types were characterized for all plasmids carrying extended-spectrum or AmpC β-lactamases. Purified plasmid DNA was transformed into DH10B cells by electroporation (Invitrogen, Van Allen Way, CA USA) under the following conditions: 1.25 kV/cm, 200 Ω, 25 μFar (*6*). Transformants were selected on Luria-Bertani agar plates supplemented with cefotaxime (1 mg/L). PCR-based replicon typing was conducted on the transformants to determine the replicon type of the plasmid by using the PBRT KIT—PCR-based replicon typing (DIATHEVA, Fano, Italy); plasmid multilocus or double-locus sequence typing (pMLST or pDLST) were used to further subtype IncI1 and IncHI2 plasmids as previously described (*7,8*). A subset of transformants (n = 16) was selected according to PFGE profile of the parental strain, replicon type of the plasmid, and antimicrobial-resistance determinant. These plasmids were subjected to S1-PFGE for accurate determination of molecular sizes (*9*). If no transformants were obtained, the chromosomal location of the extended-spectrum or AmpC β-lactamase agar plates genes was confirmed by I-*Ceu*I PFGE of total bacterial DNA, followed by Southern blot hybridization, as described (*10*).

Conjugation Experiments

The transferability of the extended-spectrum cephalosporinase-resistant phenotype by conjugation was assessed for the subset of *S. enterica* ser. Heidelberg isolates described above. Plasmid-free rifampin-resistant *E. coli* E3110 was used as a recipient strain for liquid-mating assays in a ratio of 1:1. Filter-mating assays were attempted for strains for which no transconjugants were obtained by liquid mating. For both liquid- and filter-mating assays, the donor and recipient strains in mid-exponential phase were co-incubated for 4 hours without agitation at 37° C. Transconjugants were selected on MacConkey agar supplemented with a combination of rifampin (100 mg/L) and cefotaxime (1 mg/L). Positive transconjugants were

confirmed by PCR amplification for the resistance determinant. All mating assays were conducted in triplicate. The conjugation frequency was calculated as the number of transconjugants per donor cell.

Analysis of Regions Upstream of Resistance Determinants

The association of *bla*CTX-M and *bla*CMY genes with frequently encountered insertion sequences (IS*Ecp1*, IS*CR1*, and IS*26*) was assessed for a subset of *S. enterica* ser. Heidelberg isolates (n = 17) representing each unique PFGE profile and the variation in extended-spectrum or AmpC β-lactamase gene type and its location (chromosome or plasmid replicon type). This association was investigated with PCR by using forward primers specific for IS*Ecp1*, IS*CR1,* or IS*26* and a reverse primer for *bla*CTX-M or *bla*CMY genes, as described (*11*). Subsequently, sequence analysis confirmed the amplicons obtained.

References

1. Grimont P, Weil F. Antigenic formulae of the *Salmonella* serovars. Paris: Pasteur Institute; 2007.

2. European Centre for Disease Prevention and Control. EU protocol for harmonised monitoring of antimicrobial resistance in human *Salmonella* and Campylobacter isolates. Stockholm: The Centre; 2014.

3. Dierikx CM, van Duijkeren E, Schoormans AH, van Essen-Zandbergen A, Veldman K, Kant A, et al. Occurrence and characteristics of extended-spectrum-β-lactamase- and AmpC-producing clinical isolates derived from companion animals and horses. J Antimicrob Chemother. 2012;67:1368–74. http://dx.doi.org/10.1093/jac/dks049

4. Ribot EM, Fair MA, Gautom R, Cameron DN, Hunter SB, Swaminathan B, et al. Standardization of pulsed-field gel electrophoresis protocols for the subtyping of *Escherichia coli* O157:H7, *Salmonella*, and *Shigella* for PulseNet. Foodborne Pathog Dis. 2006;3:59–67. http://dx.doi.org/10.1089/fpd.2006.3.59

5. Hunter SB, Vauterin P, Lambert-Fair MA, Van Duyne MS, Kubota K, Graves L, et al. Establishment of a universal size standard strain for use with the PulseNet standardized pulsed-field gel electrophoresis protocols: converting the national databases to the new size standard. J Clin Microbiol. 2005;43:1045–50. http://dx.doi.org/10.1128/JCM.43.3.1045-1050.2005

6. Hordijk J, Wagenaar JA, Kant A, van Essen-Zandbergen A, Dierikx C, Veldman K, et al. Cross-sectional study on prevalence and molecular characteristics of plasmid mediated ESBL/AmpC-producing *Escherichia coli* isolated from veal calves at slaughter. PLoS One. 2013;8:e65681. http://dx.doi.org/10.1371/journal.pone.0065681

7. García-Fernández A, Chiaretto G, Bertini A, Villa L, Fortini D, Ricci A, et al. Multilocus sequence typing of IncI1 plasmids carrying extended-spectrum beta-lactamases in *Escherichia coli* and *Salmonella* of human and animal origin. J Antimicrob Chemother. 2008;61:1229–33. http://dx.doi.org/10.1093/jac/dkn131

8. García-Fernández A, Carattoli A. Plasmid double locus sequence typing for IncHI2 plasmids, a subtyping scheme for the characterization of IncHI2 plasmids carrying extended-spectrum beta-lactamase and quinolone resistance genes. J Antimicrob Chemother. 2010;65:1155–61. http://dx.doi.org/10.1093/jac/dkq101

9. Barton BM, Harding GP, Zuccarelli AJ. A general method for detecting and sizing large plasmids. Anal Biochem. 1995;226:235–40. http://dx.doi.org/10.1006/abio.1995.1220

10. Liu SL, Hessel A, Sanderson KE. Genomic mapping with I-Ceu I, an intron-encoded endonuclease specific for genes for ribosomal RNA, in *Salmonella* spp., *Escherichia coli*, and other bacteria. Proc Natl Acad Sci U S A. 1993;90:6874–8. http://dx.doi.org/10.1073/pnas.90.14.6874

11. Kiiru J, Butaye P, Goddeeris BM, Kariuki S. Analysis for prevalence and physical linkages amongst integrons, ISEcp1, ISCR1, Tn21 and Tn7 encountered in *Escherichia coli* strains from hospitalized and non-hospitalized patients in Kenya during a 19-year period (1992-2011). BMC Microbiol. 2013;13:109. http://dx.doi.org/10.1186/1471-2180-13-109
